# GIS-based evaluation and spatial distribution characteristics of land degradation in Bijiang watershed

**DOI:** 10.1186/2193-1801-2-S1-S8

**Published:** 2013-12-11

**Authors:** Xiaoqing Zhao, Jinhua Dai, Jianping Wang

**Affiliations:** College of Resource Environment and Earth Science, Yunnan University, Kunming, Yunnan China

**Keywords:** Geographic information system (GIS), Land degradation, Spatial distribution, Bijiang watershed

## Abstract

Land degradation is one of the significant issues the human beings are confronted with, which has become a bottleneck of restricting the sustainable development of the regional society and economy. In order to ascertain the root causes contributed to the land degradation and characteristics of land degradation, Bijiang watershed, the most important Lead-Zinc mine area of Lanping county of Yunnan Province, was selected as the study area. One evaluation index system for land degradation that consists of 5 single factors(water-soil erosion intensity, geological disaster risk, cultivation intensity of arable land, pollution of heavy metals in soil and biodiversity deterioration) was established and 13 indicators were chosen, and the entropy method was adopted to assign weights to each single factor. By using the tools of Geographic Information System (GIS), the land degradation degree was evaluated and one spatial distribution map for land degradation was accomplished. In this study, the land of the whole watershed was divided into 4 types, including extremely-severe degradation area, severely-degraded area, moderately-degraded area and slightly-degraded area, and some solutions for ecological restoration and rehabilitation were also put forward in this study. The study results indicated that: (1) Water-soil erosion intension and pollution of heavy metals in soil have made greater contribution to the comprehensive land degradation in Bijiang watershed; (2) There is an apparent difference regarding land degradation degree in Bijiang watershed. The moderately-degraded area accounts for the most part in the region, which covers 79.66% of the whole watershed. The severely-degraded area accounts for 15.98% and the slightly-degraded regions and extremely severe degradation area accounts for 1.08% and 3.28% respectively; (3) There is an evident regularity of spatial distribution in land degradation in Bijiang watershed. The moderately-degraded areas mainly distribute in the most part of the mid-stream and down-stream, the slightly-degraded areas distribute in the mid-stream, the severely-degraded areas distribute in the upstream and south-west part of down-stream, the extremely severe degradation areas distribute in the east and middle part of the upstream; (4)From the administrative division viewpoint, the slightly-degraded areas primarily distribute in Jiancao township, Baishi town and Nuodeng town. The moderately-degraded areas distribute in Changxin township, Baofeng township, Jiancao township, Baishi town and Nuodeng town. The severely-degraded areas distribute in Jinding town, Baofeng township and Lajing town. The extremely severe degradation areas distribute in Jinding town. By connecting the spatial distribution mode for land degradation with other natural, economical elements, we drew a conclusion that the pollution in heavy metals in soil, serious water erosion and geological disasters are the main causes of the land degradation in Bijiang watershed.

## Introduction

The human-land contradiction is becoming more and more serious along with the rapid growth in population, moreover, during the last several decades, some inappropriate approaches have been adopted by human beings to exploit and utilize land driven by one-sided emphasis on socio-economic benefits, which has caused a series of land degradation issues, for example water erosion, geological disasters, decreasing productivity of arable land, pollution of heavy metals in soil and the degradation in land biological property, etc [1–3]. Land degradation has seriously damaged the eco-environmental safety of land and posed a great threat to the food security as well as exerted influence on the sustainable development of society and economy. Land degradation is not just an environmental issue but also a socio-economic one confronting the humankind [4]. Study on land degradation, especially on its mechanism, dynamic evolution, temporal and spatial distribution as well as on the countermeasures for the land ecological restoration and rehabilitation has become the hot spot of many disciplines, such as environmental science and geographic science, etc. in the twenty-first century [5]. At present, land degradation studies mainly concentrate on the driving forces of land degradation [6, 7], the measures for prevention and control of land degradation [8–11] and the methodologies for evaluating land degradation [12–14].

Bijiang River is one of the significant tributaries of Lancang River, the most famous international river in Yunnan. Jinding Lead-Zinc mine area of Lanping County of Yunan Province is well known with the reputation of "No1 in China and Second in the world" due to its great scale and rich reserves. It is located in the upstream of Bijiang watershed. A great amount of heavy metal matters, i.e. Zn, Cd, Pb, As, etc. are discharged during the process of mineral production, which has caused a serious heavy metal contamination in the water and soil, as well as a great damage to the surrounding arable land and led to frequent occurrence of geological disasters in this watershed. Besides, the erosion problem is also aggravated by the complicated topography, simplex agricultural productive patterns and intensive cultivation of farmland, etc in the watershed.

In this study, the methods of field investigation and semi-structural interview were applied to collect the first-hand data concerning about the land degradation in Bijiang watershed. In order to provide some scientific basis for the comprehensive treatment of land degradation in the watershed, one evaluation index system was established for evaluating land degradation degree and the spatial distribution map of land degradation was generated by using GIS tools. Hopefully, the study methods and evaluation index system applied in this study would provide some references for the research regarding land degradation issue in the similar areas, where the existence of mine areas have caused apparent environmental impacts.

## Materials and methods

### Profile of the study area and land degradation issues

#### Profile of the study area

The Bijiang River flows through Lanping and Yun long counties, Yunan province, Southwest China(99° 13'~99° 36' E,25° 28'~26° 41' N) from north to south. That headstream has two tributaries, the one that originates from the west of Yanlu mountain range in the northeast of Jinding Town of Lanping County is called the front Bijiang River, and the other that rises from Lvzhuping village of Lajing Town of Lanping County is called the back Bijiang River. The two rivers confluence as the whole Bijiang River in the south of Jinding town and flows into Yunlong County, and finally confluence into Lancangjiang River when reaching Gongguo Bridge.

Bijiang watershed covers seven towns(townships) from north to south, named Lajing town, Jinding town, Nuodeng town, Jiancao township, Baofeng township, Baishi town and Changxin township respectively. The total area is approximately 2440 km^2^, and about 559 km^2^ in Lanping County and 1881 km^2^ in Yunlong County. The whole length is approximately 150 km, about 30 km in Lanping County and 120 km in Yunlong County. Its average amount of the annual water production is about 8108.7 billion m^3^. This river is the main water source of agricultural irrigation, industrial water, hydropower generation and drinking water for the local people and livestock, and also plays very important roles in terms of mediating climatic and developing tourism, etc.

#### Current land degradation issues in Bijiang watershed

The main land degradation problems confronting Bijiang watershed include water and soil erosion, geological disasters, heavy metal pollution in water and soil, land damage and biodiversity deterioration, etc.

##### Water loss and soil erosion

The Departments of Environmental Protection of Lanping and Yunlong counties have paid increasing attention to the enhancement of the control and treatment of water and soil loss in recent years, but the problems remain serious. The analysis of the materials and data obtained from the field investigation shows that the area of water and soil erosion is approximately 776.75 km^2^, accounting for 31.94% of the total area of the Bijiang watershed, which has had created huge impacts on the agricultural production and environmental conservation in the watershed.

##### Geological disasters

The overexploitation of Jinding Lead-Zinc mine area in Lanping county has changed the property of rocks, the structure and the distribution of soil mass as well as has ruined the vegetation seriously, which also has led to the frequent occurrence of the mud flow, land side, land collapse land subsidence, etc.. The data from the local land administration bureau showed that during the years of 2007 and 2008, 2 large-scale mud flows and 5 serious land slides occurred around the mine area, causing 5 deaths and direct economic loss that surpass 1 million RMB. In 2009, there were 14 land slide areas, 2 land collapse areas, 8 debris flow areas and 2 land subsidence areas occurred around the mine area, and approximately 4528 hm^2^ of land was destroyed, which has posed a great threat to the life and property safety of the local people.

##### Serious heavy metal contamination in water and soil

According to the statistics from the Departments of Environmental Protection, the amount of COD discharged into Bjiang river was 920t in 2005, 1072.5t in 2006, 1412.2t in 2007 and the municipal wastewater discharged into the river was 1.2654 million tons and industrial wastewater was1.8048 million tons in 2008. According to the interviews with the local people, nearly all the aquatic creatures have gradually became extinct since the large-scale production of Jinding mine started in 2004, which has caused a serious loss of aquatic biodiversity. The quality of the water has decreased seriously and is no longer drinkable for local people and livestock. As a result, this has led to water shortage problem at the local. Moreover, it has been found that there was a remarkable degradation both in the quantity and quality of the crops in Bijiang watershed during the last several decades, because a plenty of farmland was polluted seriously by the heavy metals, such as Zn, Cd, Pb and As, etc. Most seriously, it has been reported that the heavy metals enter human bodies via food chain, which has seriously jeopardized the health of the local people, especially that of children.

##### Intensive cultivation and serious land damage

Unfortunately, driven by the direct economic benefits, local people did not pay sufficient attention to the impacts of the inappropriate cultivation patterns. Through overlaying the slope map with farmland map via GIS tools, it is found that the area of cultivated land with a slope of equal or above 25 degrees is approximately 97.2 km^2^ in 2008, accounting for 3.97% of the total area of Bijiang watershed. The exploitation of the mine area has destroyed 126.81 hm^2^ of land by 2008, including 51.35 hm^2^ of agricultural land and 75.46 hm^2^ of unused land.

### Data and methodologies

#### Data collection and processing

##### Data collection

The date collected for the study include soil distribution map, vegetation distribution map, geological map, precipitation distribution map, 1:50000 topographic map, ETM+ pseudo color landsat satellite images(2008) with a spatial resolution of 28.5 m, soil sampling data attached to accurate location that positioned by GPS, and other related socio-economic data.

##### Data processing

(1) Based on the Standard for Land Classification of P.RC. (GB/T21010-2007) and Detailed Rules for Second Land Survey in Yunnan Province(2008), the software of ERDAS IMAGES was used to interpret the landsat satellite images, and supported by GIS, the map for current situation of land utilization of Bijiang watershed was drawn. All the date were testified and modified in the field..

(2)The testing items of soil samples include the contents of Pb, Zn, As, Cd; the test method is atomic fluorescence (AFS);the test instrument is automatic double channels AFS; the test accuracy is <1.0%; the distribution map of soil samples was made based on the spatial analysis function of GIS and the spatial distribution map of heavy metals in soil was made on the support of interpolation technique of GIS.

(3) Based on the 1:50000 topographic map, the DEM and slope map were automatically generated by GIS.

#### Evaluation index system and grade criteria of land degradation

The evaluation index system for land degradation comprised of 5 single factors and 13 indicators (See Table 1) was established in accordance with the principles that data shall be representative, applicable, regionally-covered, measureable and easy to collect. Most important, the single factors and indicators chosen must adapt to the context and resource reality of Bijiang watershed. The grade standards were based on the Temporary Technique of Ecological Function Zoning of P.R.C (2002) [15].

**Table 1:** Evaluation index system and grade criteria of land degradation in Bijiang watershed

Single factors	Indicators	Land degradation grade			
		**Slight degradation**	**Moderate** **degradation**	**Severe** **degradation**	**Extremely-severe degradation**
**Water-soil erosion**	**Precipitation**	**<800**	**800 -1000**	**1000-1200**	**1200-1500**
**intensity**	**soil texture**	**Coarse sand, clay soil**	**loamy soil**	**sandy loam soil, silt clay soil, loamy clay soil**	**sandy mealy soil, mealy soil**
	**Terrain slope**	**<15**	**15-25**	**25-35**	**35-90**
	**NDVI**[16]	**[0.2,1)**	**[-0.15,0.2)**	**[-0.35,-0.15)**	**[-1,-0.35)**
The risk of geological disaster	Geological disaster density [17] (points/km^2^)	<1	1-3	3-5	>5
	hypsography degree [18]	<250	250-500	500-800	>800
	Occurrence level of geological disaster	Easy occurrence area	Moderately easy occurrence area	Highly easy occurrence area	Excessively easy occurrence area
	ratio of land with a slope above 25°	<8	8-30	30-60	>60
Biodi- versity	SIEI	>1.3	1.2-1.3	1.0-1.2	<1.0
	Important species	First-grade State protection species	Second-grade State protection species	Other State protection and provincial protection species	Other regional protection species
	Index of biological abundance [19]	>70	55-70	40-55	<40
Cultiv- ation intensity	Cultivation index of arable land	<0.35	0.35-0.6	0.6-0.8	0.8-1
	ratio of arable land with a slope above 25°	<0.02	0.02-0.03	0.03-0.05	>0.05
Pollution of heavy metals in	Content of Cd in soil [20]	<0.2	0.2-0.35	0.35-0.6	>0.6
soil	Content of Zn in soil [20]	<250	250-300	300-500	>500
	Content of Pb in soil [20]	<100	100-300	300-450	>450
	Content of As in soil [20]	<15	15-25	25-30	>30
Assigned value		3	5	7	9
Grade standard for value		2.1-4.0	4.1-6.0	6.1-8.0	>8.0

#### Evaluation methods

##### Evaluation model for single factors of land degradation

Among the five singe factors, risk of geographic disaster, cultivation intensity and biodiversity deterioration were calculated directly by using the Product Method. This method can effectively eliminate the difference between indicators in term of their dimension units and make them comparable (See Equation 1); water-soil erosion intensity was calculated by using the Product method based on USLE proposed by Wischmeier and Smith in 1978 [21] (See Equation 2); contamination index of heavy metals in soil was calculated by adopting the improved Nemoro method [22–25] (See Equation 3).1

Where Sj is single factor index; Ci is the value of indicator i; n is the number of indicators.2

Where WE is water erosion; R is rainfall erosion force; K is soil texture; LS is slope degree; C is land cover.3

Where P is pollution index of heavy metals in soil; Pimean is the average value of pollution indexes of the tested samples; Pimin is the minimum value of pollution indexes of the tested samples; n is the number of tested samples. Pi = Ci/Si,where Pi is the pollution index of pollutant i(i = Zn, As, Cd and Pb) [26]; Ci is the tested value of pollutant i; Si is the standardized value of pollutant i [20].

##### Model for the comprehensive evaluation of the land degradation

Because the single factors can only reflect the influence of each factor on land degradation, and can not reflect the comprehensive land degradation degree, the model of Multi-factors Weight Sum (See Equation 4) was therefore applied to calculate the comprehensive degree of land degradation. The spatial distribution map of comprehensive land degradation was also generated by using the weighted overlay function provided by GIS tools.4

Where LDI is the comprehensive degree of land degradation; Aj is the single factor index; Wj is the weight of each single factor.

#### Weight of the single factors

The methods for assigning weights are mainly classified into two categories: (1) Subjective weight assignment method, such as fuzzy comprehensive evaluation method, comprehensive index method, analytic hierarchy process (AHP) and efficacy factor method, etc.; (2) Objective weight assignment method, such as entropy method, factor analysis method and principle component analysis (PCA), etc. In this study, entropy method [27, 28] was applied to assign weight to the five single factors (see Table 2).

**Table 2:** Weight of the five single factors of land degradation in Bijiang watershed

Single factors	Weight
Water erosion intensity	0.29076
Risk of Geological disaster	0.19671
Cultivation intensity	0.13320
Pollution of heavy metals in soil	0.25002
Biodiversity deterioration	0.12941

#### Land degradation grade and evaluation units

The conclusion drawn upon the degree of land degradation may vary with the different baseline and classify standards applied. For instances, according to the Primary Technique for Testing Land Desertification of P.R.C., the land degradation in China was classified into 4 levels, including slight degradation, moderate degradation, intensive degradation and excessively intensive degradation; Differently, scholar Zhu (1998) classified land degradation degree into 3 levels, named slight degradation, moderate degradation and severe degradation [29]. 4-levels classification is widely applied in the international academic world [30]. In order to keep in line with the reality in Bijiang watershed and also with the international standards, the land degradation in this study was classified into 4 levels, as slight degradation, moderate degradation, severe degradation and extremely-severe degradation (see Table 3).The grade limitation for land degradation was determined according to the ultimate evaluation results and to the frequency figure of the comprehensive evaluation index of land degradation in GIS. The evaluation unit is the GRID of 30 × 30 m.

**Table 3:** Comprehensive index for land degradation evaluation and classification standard in Bijiang watershed

Land degradation grade	Slight degradation	Moderate degradation	Severe degradation	Extremely-severe degradation
Comprehensive index	0[3]	[3, 5]	[5, 7]	[7, 10]

## Results

**Figure 1:**
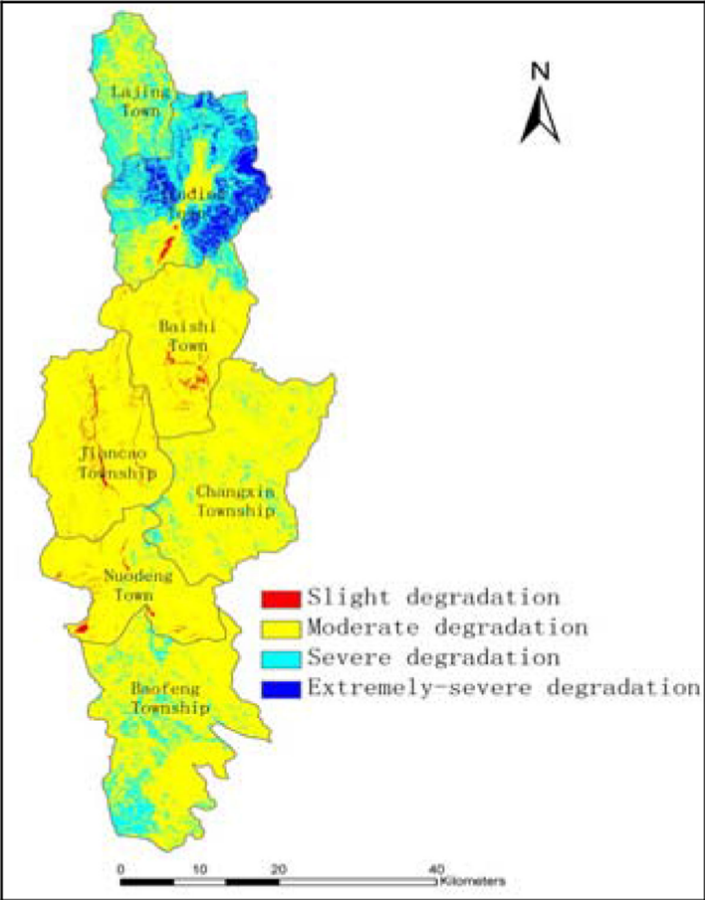
**Map for the spatial distribution of land degradation evaluation in Bijiang watershed**.

By overlaying the comprehensive evaluation map of land degradation with the administrative map of Bijiang watershed, the administrative-spatial map for land degradation was created (See Figure 1). The area of each degradation grade (See Table 4) and distribution in each administrative town were calculated by using the spatial statistical function of GIS (See Table 5).

**Table 4:** Statistics for comprehensive evaluation of land degradation in Bijiang watershed

Degradation grade	Area/hm^2^	Rate
Slight degradation	2636.49	1.08%
Moderate degradation	194366.97	79.66%
Severe degradation	38987.01	15.98%
Extremely-severe degradation	8009.64	3.28%

**Table 5:** Statistics of land degradation in each town (township) in Bijiang watershed

Towns(T-ownships)	Slight degrada-tion/hm^2^	Moderate degradation /hm^2^	Severe degradat-ion/hm^2^	Extremely-severe degradation/hm^2^
Lajing town	0	10469.00	7760.00	14.00
Jinding town	231.00	10542.00	18543.00	7962.00
Nuodeng town	564.00	25027.02	390.15	0
Jiancao township	1057.00	35171.91	0	0
Changxin township	0	43032.60	2731.68	0
Baishi town	778.00	29873.00	1523.07	31.68
Baofeng township	1.00	40158.18	8034.30	0

There is a notable difference in land degradation degree and apparent regularity regarding the spatial distribution of the land degradation in Bijiang watershed. The relevant characteristics and some corresponding countermeasures for land degradation preventing and control are proposed as follows:

### Slight degradation area

The areas with slight degradation takes up the least area, just accounting for 1.08% of the whole watershed, which mainly distribute in the middle stream from a spatial view and in Jiancao township, Baishi town and Nuodeng town from the administrative regionalization point. The soil condition is very good in these areas so the degradation issues, i.e. water and soil erosion and geological calamities, etc. area are rarely occur even under the impacts of various human activities. In these regions, prevention has top priority and the protection of the forestry resources should be emphasized., local environmental protection departments may promote recycle economy and establish some the ecological demonstration areas regarding land and natural reserves so as to achieve the coordinated and sustainable development of land eco-environment and society and economy.

### Moderate degradation area

The areas with moderate degradation take up the most area, accounting for 79.66% of the total area of the watershed, which mainly distribute in the most part of mid-stream and down-stream from a spatial view and in Changxin township, Baofeng township, Jiancao township, Baishi town and Nuodeng town from the administrative regionalization point. The problems of water erosion and geological disasters, etc. are easily happen when disturbed by human activities. The problems would be becoming more and more serious if the timely countermeasures were not applied, which would largely affect the land eco-security of the whole watershed. It is suggested in this study that the local land administrative departments should formulate one land use planning in advance to prevent the occurrence of these problems.

### Severe degradation area

The areas with severe degradation take up the second largest area in Bijiang watershed, accounting for 15.98% of the total area of the watershed, and mainly distribute in the upstream and southwest of down-stream from a spatial view and in Jinding town, Baofeng township and Lajing town from the administrative regionalization point. The land eco-environment has been seriously disturbed by human beings and most land is not suitable for intense land utilization as well as other development approaches. It is highly recommended that the active countermeasures of converting the farmland into forestry land or grassland should be taken immediately and the regulations and rules of log ban should be implemented strictly in order to protect and restore the land ecosystem.

### Extremely-severe degradation area

The areas with extremely-severe degradation is small, accounting for 3.28% of the total area of the watershed, which mainly distribute in the upstream and middle stream from a spatial view and in Jinding town from the administrative regionalization point. The main causes for the serious land degradation in the town are the pollution of heavy metals in soil, the water erosion and the geological disasters. So the necessary bio-engineering technologies are needed to reduce the content of heavy metals in soil. The sloping land protection project measures shall be taken to increase the coverage of forestry in order to reduce the occurrence of geological disasters and water erosion.

## Conclusions and discussions

### Conclusions

(1) In the study, the product method and the model of Multi-factors Weight Sum were adopted to make comprehensive evaluation of land degradation on the support of spatial technique provided by GIS. The results showed that the evaluation methods and models applied in this study are practicable. The evaluation results not only reflect the land degradation degree quantitatively, but also reveal the spatial distribution characteristics of land degradation accurately. The study results prove to be scientific and applicable through field check, which will provide some valuable references for the comprehensive control and treatment of land degradation in Bijiang watershed.

(2) There is an apparent difference regarding land degradation degree in different areas of the Bijiang watershed due to the influences from some natural factors, such as terrain, hydrological geology and rainfall, etc. and some inappropriate land use approaches by humankind, such as deforestation, intensified cultivation of farmland and overexploitation of Lead-Zinc mine area, etc. The moderate degradation areas make up the most part of the watershed, accounting for 79.66%, major portion of which distribute in the mid-stream and down-stream and small portion of which distribute in the central and northwest up-stream.

(3) Among the five single factors, the weights of erosion intensity and pollution of heavy metals in soil are up to 0.29062 and 0.25002 respectively, which suggests that these two single factors have made greater contribution to the comprehensive land degradation. The study showed that the overexploitation of Lead-Zinc mine area has led to a serious land damage and pollution of heavy metals in soil, which has seriously destroyed the land ecological function and caused a great decrease in land quality.

### Discussions

(1) Land degradation is a dynamic process. At the same time, the static method was applied in the study to evaluate the current situation of land degradation in Bijiang watershed. The analyzing results quantitatively reflect the present situation and spatial distribution characteristics of land degradation in the watershed. More attempts regarding the dynamic evaluation of land degradation will be more stressed in the future study.

(2) One of the significant characteristics of land degradation in Bijiang watershed is the contamination of heavy metals in soil caused by the exploitation of Lanping Lead-Zinc mine area, which has made a great contribution to the land degradation. So contamination of heavy metals in soil was selected as an important evaluation single factor in the study. It is needed to point out that and the index system used in this study is very much context-based and just suitable for the watershed where mine areas locate.
